# Structural determinants of inhibition of *Porphyromonas gingivalis* gingipain K by KYT-36, a potent, selective, and bioavailable peptidase inhibitor

**DOI:** 10.1038/s41598-019-41354-3

**Published:** 2019-03-20

**Authors:** Tibisay Guevara, Arturo Rodríguez-Banqueri, Anna M. Lasica, Miroslaw Ksiazek, Barbara A. Potempa, Jan Potempa, F. Xavier Gomis-Rüth

**Affiliations:** 1 Proteolysis Lab, Structural Biology Unit, “María de Maeztu” Unit of Excellence, Molecular Biology Institute of Barcelona (CSIC), Barcelona Science Park, Helix Building, c/Baldiri Reixac, 15-21, 08028 Barcelona, Catalonia Spain; 2 Department of Bacterial Genetics, Faculty of Biology, University of Warsaw, ul. Miecznikowa 1, 02-096 Warszawa, Poland; 3 Department of Oral Immunology and Infectious Diseases, University of Louisville School of Dentistry, 501 South Preston Street, Louisville, KY 40202 USA; 4 Department of Microbiology, Faculty of Biochemistry, Biophysics and Biotechnology, Jagiellonian University, ul. Gronostajowa 7, 30-387 Kraków, Poland

## Abstract

*Porphyromonas gingivalis* is a member of the dysbiotic oral microbiome and a “keystone pathogen” that causes severe periodontal disease, which is among the most prevalent infectious diseases. Part of the virulence factors secreted by *P. gingivalis* are the essential cysteine peptidases gingipain K (Kgp) and R (RgpA and RgpB), which account for 85% of the extracellular proteolytic activity of the pathogen and are thus prime targets for inhibition. We report the high-resolution (1.20 Å) complex structure of Kgp with KYT-36, a peptide-derived, potent, bioavailable and highly selective inhibitor, which is widely used for studies *in vitro*, in cells and *in vivo*. Sub-nanomolar inhibition of Kgp is achieved by tight binding to the active-site cleft, which is covered for its sub-sites S_3_ through S_1_’ under establishment of nine hydrophobic interactions, 14 hydrogen bonds and one salt bridge. In addition, an inhibitor carbonyl carbon that mimics the scissile carbonyl of substrates is pyramidalized and just 2.02 Å away from the catalytic nucleophile of Kgp, C^477^Sγ. Thus, the crystal structure emulates a reaction intermediate of the first nucleophilic attack during catalysis of cysteine peptidases. The present study sets the pace for the development of tailored next-generation drugs to tackle *P. gingivalis*.

## Introduction

The human oral microbiome is extraordinarily diverse and includes phages, viruses, archaea, bacteria, fungi, and protozoa^1^. Bacteria are represented by ~1000 different species at 10^8^–10^9^ bacteria per mL saliva or mg dental plaque, which makes the oral microbiome second only to the colon microbiome in complexity^2^. Oral bacteria mainly belong to the phyla *Actinobacteria*, *Bacteroidetes*, *Firmicutes*, *Proteobacteria*, *Spirochaetes*, *Synergistetes* and *Tenericutes*, and they divide into commensal and dysbiotic. While the former are usually beneficial to the host, the latter are associated with disease due to changes in microbiome composition and functional activities^3^. Dysbiotic bacteria are mostly Gram-negative and anaerobic, and they are responsible for two of the most widespread human diseases: dental caries and periodontal disease (PD). These are not classical infectious diseases originated by single pathogens but have polymicrobial origins and result from a combination of microbiota relationships, host susceptibility, and environmental factors, such as smoking and diet^1,4^. In particular, PD is the sixth most prevalent disabling health condition and affects an estimated ~750 million people worldwide^5^. It causes alveolar bone resorption, formation of deep periodontal pockets, and tooth loosening, and is epidemiologically associated with several systemic diseases including atherosclerosis, diabetes and cardiovascular conditions^6^. PD derives from an exacerbated inflammatory response to normal microbiota triggered by the presence of dysbiotic species including *Aggregatibacter* (formerly *Actinobacillus*) *actinomycetemcomitans*, *Fusobacterium nucleatum*, *Prevotella intermedia*, *Treponema denticola*, *Tannerella forsythia* and *Porphyromonas gingivalis*. For many years, the latter three species were englobed in the “red complex,” which substantially contributes to the subgingival biofilm and plaque and is intimately associated with severe forms of PD^7^. Among these species, *P. gingivalis* is a “keystone pathogen,” which converts other benign members of the biofilm into pathobionts and causes aggressive damage to periodontal tissues^8^. To this aim, it employs an armamentarium of virulence factors, which further contribute to pathogenesis by deregulating immune and inflammatory responses in the host.

*P. gingivalis* virulence factors include peptidases, which break down proteins within infected tissues, thus nourishing bacteria and facilitating their dissemination and host colonization^9^. Peptidases also dismantle host defenses and outcompete bacterial competitors within periodontal pockets^10^. The most relevant are the cysteine peptidases gingipain K (*alias* Kgp) and R (RgpA and RgpB), which cleave proteins and peptides after lysines and arginines, respectively^11^. They are translocated from the periplasm across the outer membrane layer to the extracellular space through a type-IX secretion system, which consists of at least 18 proteins, some of which are engaged in post-translational modification of cargo proteins^12,13^. The signal for translocation is a C-terminal domain conserved across cargos, which in RgpB adopts an immunoglobulin-like fold encompassing seven antiparallel β-strands organized in a β-sandwich^14^.

Gingipains are detected at concentrations exceeding 100 nM^15^ in gingival crevicular fluid from *P. gingivalis*-infected periodontitis sites, where they account for 85% of the total extracellular proteolytic activity of the bacterium^16,17^. Kgp, which is responsible for most of this activity^18^, is a 1723/1732-residue multidomain enzyme encompassing an N-terminal signal peptide, a pro-domain for latency, a caspase-like cysteine peptidase catalytic domain (CD), an immunoglobulin superfamily-like domain (IgSF), between three and five hemagglutinin-adhesion domains, and the C-terminal domain for type-IX secretion^19^. Kgp degrades connective tissue and plasma proteins, for example heme- and hemoglobin-transporting proteins, fibrinogen, fibronectin, plasma kallikrein, immunoglobulins, as well as peptidase inhibitors, thus causing vascular permeability and bleeding^19,20^. Kgp is indispensable for bacterial survival and the outcome of PD^16,18^, and has thus been hailed as a prime target for the development of novel drugs to treat PD^19,21,22^. This is of particular importance given that the current standard treatment of PD includes mechanical debridement and the widespread use of antibiotics and disinfectants, which have serious adverse effects due to toxicity and the development of bacterial resistance. Moreover, this treatment does not guarantee disease eradication^23^.

**Figure 1:**
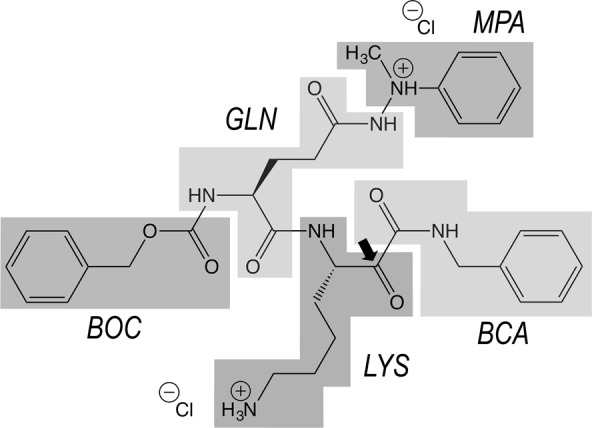
Chemical structure of KYT-36. The inhibitor, with IUPAC name benzyl-*N*-[(2*S*)-1-[[(3*S*)-7-amino-1-(benzylamino)-1,2-dioxoheptan-3-yl]amino]-5-(2-methyl-2-phenylhydrazinyl)-1,5-dioxopentan-2-yl]carbamate, consists of benzyloxycarbonyl (BOC), L-glutaminyl (GLN), methylphenylamino (MPA), L-lysinyl (LYS), and benzylcarbamoyl (BCA) moieties. A black arrow indicates the carbonyl mimicking the scissile carbonyl of a substrate. The molecular mass of the dichloride salt is 703.7 Da.

While a lot of effort has been dedicated lately to phylogenetic associations and meta-omics of the oral microbiome^24,25^, molecular and functional studies to discover valid biomarkers of oral pathophysiology, understand host–microbiome interactions, and develop novel drugs have largely been neglected^1^. An exception is the drug precursor candidate KYT-36 (Fig. 1), a peptide-derived, small-molecule inhibitor developed in 2004 in the laboratory of Kenji Yamamoto^26^. It is very specific for and potent against Kgp (*K*_i_ ≈ 10^−10^ M). Together with inhibitor KYT-1, which specifically tackles RgpA and RgpB, it strongly inhibited degradation of host proteins in culture supernatants and abolished thriving of *P. gingivalis* in cell cultures and in periodontal pockets *in vivo*. Moreover, it prevented Kgp-triggered vascular permeability in guinea pigs, i.e. demonstrating its efficacy against bacterial virulence *in vivo*, with no toxicity effects at the doses tested^19^. Based on these properties, the molecule and its derivatives are subject of patents by Cortexyme, Inc. for the therapeutic treatment of *P. gingivalis* (US20160096830A1, US2017014468A1 and WO2017201322A1) and by others (JP2010270061A and JP4982908B2). KYT-36 is currently distributed by at least four companies (Peptides International, www.pepnet.com; Pepta Nova, peptanova.de; MyBioSource, www.mybiosource.com; and Peptide Institute, www.peptide.co.jp) and has been used for years as the Kgp inhibitor of reference for studies *in vitro*, in cells and *in vivo* (see^21,22,27^ for examples).

Whilst the efficacy of KYT-36 is well established, no information is available on its chemical mechanism of inhibition. This information is provided by three-dimensional structural studies, which are part of *rational drug design* strategies^28,29^. To this aim, we recently determined the crystal structure of the CD and IgSF domains of Kgp^30^ and of their zymogenic complex with the pro-domain^31^. These results revealed the mechanisms of action and latency of this peptidase. Here, we analyzed the crystal structure of Kgp from *P. gingivalis* strain W83 in complex with KYT-36 to very high resolution (1.20 Å). This is the first complex structure of the major proteolytic virulence factor of the periodontal pathogen reported with a drug or lead compound.

## Results and Discussion

### Structure of the Kgp catalytic domain

**Figure 2:**
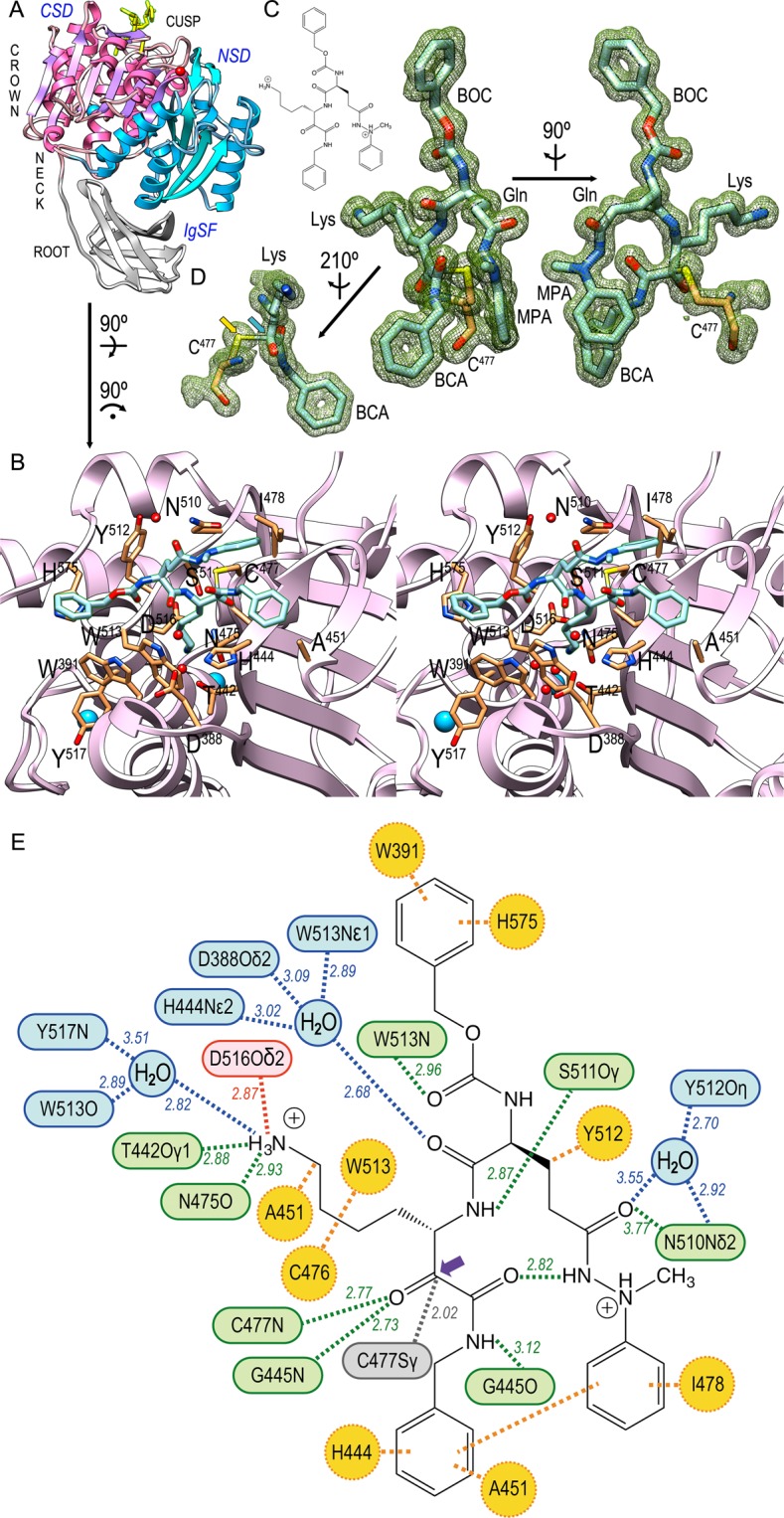
Interactions of the Kgp·KYT-36 complex. (**A**) Ribbon plot of Kgp, which mimics a tooth, whose crown encompasses the cusp in the top and consists of the NSD (blue ribbon) and CSD domains (magenta ribbon). Domain IgSF (grey ribbon) features the tooth root. KYT-36 is displayed as yellow sticks for reference. (**B**) Close-up of the tooth cusp encompassing the active site. The cleft runs from left (non-primed sub-sites) to right (primed sub-sites). Only the CSD is displayed as a plum ribbon for clarity. Kgp residues relevant for the complex are shown for their side chains (carbons in sandy brown) and labeled. The proposed catalytic triad is C^477^, H^444^ and D^388 ^^30^. Solvent molecules and structural sodium cations are depicted as red and blue spheres, respectively. KYT-36 is shown as a stick model with carbons in light blue. (**C**) Structure of KYT-36 and Kgp catalytic residue C^477^ superposed with a (2m*F*_obs_-D*F*_calc_)-type Fourier map contoured at 0.8σ (*left*) and after a 90°-rotation (*right*). The five moieties of the inhibitor (see Fig. 1) are labeled. The inset in the top left depicts the chemical structure of the inhibitor for reference. (**D**) Detail of (C, *left*) after reorientation depicting the pseudo-covalent bond (2.02 Å) between C^477^Sγ (yellow arrow) and the carbonyl carbon of LYS (blue arrow), which mimics the scissile carbonyl carbon of a substrate and is pyramidalized. (**E**) Scheme with the average distance values of direct (green) and solvent-mediated (blue) hydrogen bonds, salt bridges (red), hydrophobic interactions (orange), and the pseudo-covalent bond between the LYS carbonyl carbon (purple arrow) and catalytic C^477^Sγ (grey).

The Kgp fragment analyzed encompassed domains CD (residues D^229^-P^600^) and IgSF (K^601^-P^683^). Taken together, these domains form an elongated structure that resembles a tooth: the CD forms the crown with the cusp at its top, and the IgSF, which is a six-stranded antiparallel open β-barrel, shapes the root (see Fig. 2A). The CD is subdivided into an N-terminal subdomain (NSD; D^229^-K^375^) and a C-terminal subdomain (CSD; S^376^-P^600^), which are laterally attached to each other. Each of these subdomains is an α/β/α-sandwich consisting of a central β-sheet flanked by α-helices on either side. In NSD, the sheet is four-stranded and parallel; in CSD, it is six-stranded and parallel for all strands except the outermost strand at the interface with NSD, which is antiparallel to all other strands. In this way, the overall structure spans a central pseudo-continuous ten-stranded β-sheet. The NSD further contains two and three helices on either side of the sheet, respectively, *plus* an inserted β-ribbon and a calcium-binding site with structural functions. The CSD contains five and four helices on either side of the sheet, respectively, *plus* a β-ribbon and two sodium-binding sites. A second calcium site is found at the NSD-CSD interface. For further structural details on the general architecture of Kgp, see^30^.

The active-site cleft of Kgp is found at the tooth cusp, on the CSD surface (Fig. 2A,B). As common in α/β-hydrolase-enzymes, residues engaged in substrate binding and catalysis come from loops that link strands of the central β-sheet on its the C-terminal edge^32^. As found in other cysteine peptidases^33^, Kgp probably contains a catalytic triad (C^477^, H^444^ and D^388^), which may form a charge-relay system for catalysis^19,30,34^. Atom C^477^Sγ acts as the nucleophile that attacks the scissile carbonyl carbon of substrates, which in a first step proceeds over a covalent tetrahedral reaction intermediate to an acyl-enzyme thioester complex with concomitant release of the amine reaction product^35^. In a second step, the covalent acyl-enzyme is hydrolyzed by a solvent molecule to release the acyl reaction product. In Kgp, substrates are bound with a lysine intruding the specificity pocket (sub-site S_1_) of the active-site cleft (for substrate and enzyme sub-site nomenclature, see^36^). The bottom of the specificity pocket leads to an internal water channel, which extends across the CSD to the opposite outer surface of the subdomain^30^.

### The Kgp·KYT-36 complex

The complex structure was determined to very high resolution (1.20 Å; Fig. 2C,D), which enabled us to unambiguously assign the molecular determinants that cause sub-nanomolar inhibition of Kgp (Fig. 2B,E). KYT-36 is a L-peptide-derived molecule that mimics a substrate binding in extended conformation to cleft sub-sites **S**_**3**_, **S**_**2**_, **S**_**1**_ and **S**_**1**_**’** (Fig. 2B). It can be divided into five moieties: BOC, GLN, MPA, LYS and BCA (Fig. 1).

The BOC benzyl group nestles in a hydrophobic pocket created by the side chains of H^575^ and W^391^, which together with Y^512^ and W^513^ create a shallow S_3_ sub-site in Kgp. The BOC carbonyl, which imitates the eponymous group of a substrate residue in position P_3_, is hydrogen-bonded to W^513^N (Fig. 2E). Downstream moiety GLN is in S_2_ and thus protrudes into the bulk solvent. Its side chain is folded towards the primed side of the cleft and its main-chain carbonyl establishes solvent-mediated hydrogen-bonds with H^444^Nε2, D^388^Oδ2, and W^513^Nε1. The aliphatic part of its side chain interacts with Y^512^ and its side-chain carboxamide performs an intramolecular hydrogen bond *via* the nitrogen with the carbonyl of the downstream BCA moiety. In addition, the carboxamide oxygen makes a direct and a solvent-mediated hydrogen bond with N^510^Nδ2 and Y^512^Oη, respectively. The aromatic phenyl ring of the MPA moiety hydrophobically interacts with I^478^—which explains why this residue has disallowed main-chain conformation angles—and, intramolecularly, with the benzyl group of the BCA moiety.

The LYS group of KYT-36 simulates a substrate residue in P_1_ and thus matches the specificity of the enzyme^19^. Its side chain penetrates the specificity pocket and its aliphatic part is pinched between W^513^, A^451^ and C^476^ through hydrophobic interactions. The terminal ε-amino group is tetrahedrally bound by D^516^Oδ2 through a salt bridge, by N^475^O and T^442^Oγ1 through direct hydrogen bonds, and by Y^517^N and W^513^O through hydrogen bonds mediated by a solvent molecule (Fig. 2E). The interactions made by T^442^ and H^444^ to bind the inhibitor also explain why intermediate residue A^443^ has disallowed main-chain conformation angles. Further downstream, the BCA moiety possibly occupies the **S**_**1**_**’** sub-site and its amide nitrogen is hydrogen-bonded to G^445^O. In addition, the benzyl group establishes hydrophobic interactions with A^451^ and H^444^.

The LYS carbonyl emulates the scissile carbonyl of a substrate and its oxygen is tightly bound by C^477^N and G^445^N, which play the role of an oxyanion hole^35^ in Kgp to stabilize the tetrahedral reaction intermediate. The carbonyl carbon is just 2.02 Å apart (2.00 Å and 2.03 Å in the two Kgp molecules A and B found in the asymmetric unit of the crystal, respectively) from catalytic C^477^Sγ, which is roughly perpendicular to the carbon and its three bound atoms (Fig. 2D). This distance is larger than a standard aliphatic single C-S bond (1.82 Å;^37^) and the covalent bond found in the 1.75 Å-resolution structure of Kgp with a lysylmethyl group (1.84 Å; Protein Data Bank access code [PDB] 4RBM;^30^). However, the distance is shorter than that reported for reaction-intermediate mimics of serine endopeptidases in complex with protein inhibitors (2.6 Å for the complex between trypsin and bovine pancreatic trypsin inhibitor;^38^) and also than the sum of the van-der-Waals radii of carbon and sulfur (3.50 Å;^39^). In addition, the inhibitor carbonyl carbon is pyramidalized, i.e. not coplanar with its three bound atoms but shifted towards a tetrahedral configuration. This is reflected by angles C^477^Sγ-LYS(C)-LYS(O), C^477^Sγ-LYS(C)-LYS(Cα) and C^477^Sγ-LYS(C)-BCA(C) spanning on average 109.9°, 103.4° and 99.9°, respectively, instead of 90°. Thus, the present structure simulates a state immediately previous to formation of the tetrahedral reaction intermediate of the nucleophilic addition.

### Conclusions

The complex structure of Kgp with its specific inhibitor KYT-36 revealed that the sub-nanomolar inhibition exerted by the inhibitor is based on 24 intermolecular interactions and the fact that the active-site cleft of the enzyme is blocked from sub-sites S_3_ to S_1_’. The side chain of inhibitor moiety LYS penetrates the S_1_ pocket like a substrate and makes four hydrogen bonds and a salt bridge, in addition to hydrophobic interactions with three protein residues.

The complex is also a valid model for the state preceding the formation of the tetrahedral reaction intermediate of the nucleophilic attack of C^477^Sγ onto the scissile carbonyl carbon during catalysis. This is reminiscent of structures of complexes between serine endopeptidases and protein inhibitors. In either case, the distances between the catalytic nucleophile and the scissile-carbonyl-carbon-mimic are larger than a regular bond but too short for a van-der-Waals interaction. Moreover, the carbon is pyramidalized, i.e. in a state preceding the tetrahedral intermediate.

Finally, the present data will foster the development of novel specific drugs against a major virulence factor of *P. gingivalis*, which may add to the locally-applied therapeutic agents currently used for PD as adjuncts to non-surgical therapy^22^. These adjuncts include doxycycline and minocycline, which are tetracycline antibiotics that inhibit host matrix metalloproteinases at doses low enough not to have antimicrobial activity. In this way, they do not select for antibiotic resistance within bacteria^40^. The potential of KYT-36 to contribute to such a development was demonstrated recently by KYT-41. This is a further development of KYT-36 and KYT-1, which potently and selectively blocks both Kgp (*K*_i_ = 2.7 × 10^−10^ M) and RgpA/B (*K*_i_ = 4.0 × 10^−8^ M), and shows therapeutic potential in guinea pig and dog models^21^.

## Experimental Procedures

### Protein production and complex formation

A Kgp construct spanning the CD and IgSF domains from *P. gingivalis* strain W83 (sequence D^229^-P^683^; UniProt (UP) entry Q51817) and a C-terminal His_6_-tag was purified from culture medium of *P. gingivalis* mutant strain ABM1 by affinity chromatography on Nickel-Sepharose beads as previously described^41,42^. The resulting sample was first incubated with *N*α-tosyl-L-lysinylchloromethane (Sigma) prior to elution from the beads to avoid autolysis and then with excess of KYT-36 (purchased from Peptide International, KY, USA). The final complex was concentrated to ~10 mg/ml in 5 mM Tris·HCl pH 8, 150 mM sodium chloride, 0.02% sodium azide, 1 mM 1,4-dithiothreitol (DTT) for crystallization.

### Crystallization and diffraction data collection

Crystallization assays were performed by the sitting-drop vapor diffusion method. Reservoir solutions were prepared with a Tecan robot and 100-nL crystallization drops were dispensed on 96 × 2-well MRC nanoplates (Innovadyne) by a Phoenix nanodrop robot (Art Robbins) or a Cartesian Microsys 4000 XL (Genomic Solutions) robot at the IBMB-IRB joint Automated Crystallography Platform at Barcelona Science Park. Plates were stored in Bruker steady-temperature crystal farms at 4 °C or 20 °C. Successful conditions were scaled up to the microliter range in 24-well Cryschem crystallization dishes (Hampton Research). The best Kgp·KYT-36 complex crystals were obtained at 20 °C with protein solution and 20% polyethylene glycol 8000, 0.1 M HEPES pH 7.5 as reservoir solution from 1 μL: 1 μL drops. Crystals were cryo-protected by immersion in harvesting solution containing reservoir solution *plus* 20% glycerol. Diffraction data were collected at 100 K from liquid-N_2_ flash cryo-cooled crystals (Oxford Cryosystems 700 series cryostream) on a Pilatus 6 M pixel detector (Dectris) at beam line XALOC^43^ of the ALBA synchrotron in Cerdanyola (Catalonia, Spain). These data were processed with programs XDS^44^ and XSCALE^45^, and transformed with XDSCONV to formats suitable for the CCP4 suite of programs^46^. Given that cell constants a and b were very similar (86.66 Å and 87.05 Å, respectively), initial indexation suggested a tetragonal setting. This was proven wrong during integration and merging of the reflections, which revealed that the crystals actually belonged to a primitive orthorhombic space group with two peptidase·inhibitor complexes per asymmetric unit.

### Structure solution and refinement

The structure of Kgp·KYT-36 was solved by likelihood-scoring molecular replacement with the PHASER^47^ program using the coordinates of the protein part of Kgp crystallized in a different unit cell (PDB 4RBM;^30^) and diffraction data processed to 1.25 Å resolution. Two solutions were obtained at final Eulerian angles (α, β, γ, in °) 188.9, 71.0, 312.8 and 287.3, 71.3, 313.9; and fractional cell coordinates (x, y, z) 0.472, −0.103, 0.293 and −0.209, −0.295, 0.529, respectively. The initial values for the rotation/translation function Z-scores were 7.4/8.0 and 7.7/9.0, respectively, and the final log-likelihood gain was 62,873. These calculations revealed that P2_1_2_1_2_1_ was the correct space group. Subsequently, an automatic tracing step with ARP/wARP^48^ yielded a model, which was completed through successive rounds of manual model building with the COOT program^49^ and crystallographic refinement with the PHENIX^50^ and BUSTER/TNT^51^ programs, which included TLS refinement. In the final stages, anisotropic B-factor and alternate occupancy refinement was performed with BUSTER/TNT using data reprocessed to 1.20 Å resolution (see Table 1 for data processing statistics). The final model contained residues D^229^-G^681^, two calcium and two sodium cations, two unknown atoms/ions (UNK), and one KYT-36 moiety for each of the two Kgp molecules in the asymmetric unit. Further one DTT, six glycerols, two HEPES, and 1,580 solvent molecules completed the final model. The HEPES molecules were only tentatively assigned based on poor density and show two positions. Three residues of each molecule (A^443^, I^478^ and I^576^) were in disallowed regions of the Ramachandran plot but were unambiguously resolved in the final Fourier map. Three proline residues were found in *cis* conformation (P^241^, P^424^, and P^453^). Table 1 provides refinement and model validation statistics.

**Table 1:** Crystallographic data.

Dataset	Kgp·KYT-36
***Data processing***	
Space group	P2_1_2_1_2_1_
Cell constants (a, b, c, in Å)	86.67, 87.05, 129.21
Wavelength (Å)	0.97949
No. of measurements/unique reflections	2,922,120/290,672
Resolution range (Å)	72.2 – 1.20 (1.27 – 1.20)^a^
Completeness (%)	95.8 (75.2)
R_merge_	0.065 (0.726)
R_meas_/CC^1/2^	0.068 (0.800)/1.000 (0.843)
<I/σ(I)> of unique reflections after merging	19.6 (2.9)
B-Factor (Wilson) (Å^2^)/Aver. Multiplicity	15.9/10.1 (5.2)
***Structure refinement***	
Resolution range used for refinement (Å)	72.2 – 1.20
No. of reflections used (test set)	289,551 (1120)
Crystallographic R_factor_ (free R_factor_)	0.146 (0.148)^b^
No. of protein residues + atoms/solvent molecules/ligands^c^	906 + 7,048/1,580/2 K36, 4 Ca^2+^, 4 Na^+^, 4 UNK, 1 DTT, 6 GOL, 2 EPE
Correlation coefficient F_obs_-F_calc_	0.962^b^
*Rmsd* from target values^b^	
bonds (Å)/angles (°)	0.012/1.17
Average B-factors (Å^2^) (all// molec. A/B)	16.3//13.2/13.3
Overall anisotropic B-value (B11, B22, B33, in Å^2^)	1.04, −3.67, 2.64
All-atom contacts and geometry analysis^d^	
Protein residues in favored regions/outliers/all residues	915^e^ (97%)/6^f^/947^e^
Protein residues with outlying rotamers/bonds/angles/chirality/planarity	2/0/0/0/0
All-atom clashscore	2.7

^a^Data processing values in parenthesis are for the outermost resolution shell. ^b^According to the final BUSTER/TNT refinement step. ^c^K36, KYT-36; DTT, (2S,3S)-1,4-bis(sulfanyl)butane-2,3-diol; GOL, glycerol; UNK, unknown atoms/ions; and EPE, 4-(2-hydroxyethyl)-1-piperazineethanesulfonic acid (HEPES.). ^d^wwPDB X-ray Structure Validation Report. ^e^Including residues with atoms in two positions. ^f^All outliers are unambiguously resolved in the final Fourier map.

Once the structure was solved, the two molecules in the asymmetric unit were found to be related by a pure non-crystallographic twofold parallel to (1 1 0). Upon superposition of the protein moieties, the two inhibitor molecules perfectly matched and were engaged in crystal contacts with segment Y^389^-Q^394^ from the non-crystallographic symmetry mate. These contacts are very similar but not identical, which might actually have given rise to rupture of the tetragonal symmetry suggested by the indexation procedure.

### Miscellaneous

Ideal coordinates and parameters for crystallographic refinement of KYT-36 were obtained from the PRODRG server^52^. Structure figures were prepared with the CHIMERA program^53^. The model was validated with the wwPDB Validation Server (https://www.wwpdb.org/validation;)^54^. The final coordinates of *P. gingivalis* Kgp·KYT-36 are deposited with the PDB at www.pdb.org (access code 6I9A).
